# Pegiviruses and Coronavirus: Biomolecular Prevalence and Phylogenetic Analysis of Strains Detected in Italian Horse Populations

**DOI:** 10.3390/v17081076

**Published:** 2025-08-02

**Authors:** Ida Ricci, Francesca Rosone, Giulia Pacchiarotti, Giuseppe Manna, Antonella Cersini, Andrea Carvelli, Davide La Rocca, Elisa Cammalleri, Roberta Giordani, Silvia Tofani, Raffaella Conti, Pasquale Rombolà, Roberto Nardini, Carlo Alberto Minniti, Reno Caforio, Boris Linardi, Maria Teresa Scicluna

**Affiliations:** 1 Istituto Zooprofilattico Sperimentale del Lazio e della Toscana “M. Aleandri”, 00178 Rome, RM, Italy; ida.ricci@izslt.it (I.R.); francesca.rosone@izslt.it (F.R.); giuseppe.manna@izslt.it (G.M.); antonella.cersini@izslt.it (A.C.); andrea.carvelli@izslt.it (A.C.); davide.larocca@izslt.it (D.L.R.); elisa.cammalleri@izslt.it (E.C.); roberta.giordani@izslt.it (R.G.); silvia.tofani@izslt.it (S.T.); raffaella.conti@izslt.it (R.C.); pasquale.rombola@izslt.it (P.R.); roberto.nardini@izslt.it (R.N.); teresa.scicluna@izslt.it (M.T.S.); 2 Dipartimento per l’Organizzazione Sanitaria e Veterinaria-Comando Generale dell’Arma dei Carabinieri, 00197 Rome, RM, Italy; carlo.minniti@carabinieri.it; 3 Esercito Italiano, Comando Sanità e Veterinaria, Reparto Veterinaria, 00162 Rome, RM, Italy; reno.caforio@esercito.difesa.it (R.C.); boris.linardi@esercito.difesa.it (B.L.)

**Keywords:** *Equine Pegivirus* (*caballi* and *equi*), *Equine coronavirus*, Italy

## Abstract

Equestrian sports play a significant economic role in the horse industry. In recent years, numerous equine viruses have emerged, among which are equine Pegiviruses and the re-emerging Equine coronavirus (ECoV). These viruses are distributed globally and primarily cause subclinical infections with unknown morbidity, even if ECoV can occasionally induce febrile and diarrheic episodes. To broaden the data on the Italian equine population, a study was conducted to assess their prevalence in two distinct horse populations belonging to the Carabinieri Corps (CC) and the Italian Army (IA) of the Italian Armed Forces (IAF). Samples consisted of blood serum and rectal swabs of 436 horses collected within the national surveillance program for equine infectious anemia and gastrointestinal parasite monitoring and analyzed for *Pegivirus* (*caballi* and *equi*) and ECoV by Real-Time RT PCR. The prevalence detected were 6.56% and 3.53%, respectively, for *Pegivirus caballi* and *equi* for the IA, while for the CC, they were 10.13% and 0.84%. Only one sample tested positive for *ECoV* belonging to a horse of the CC. Phylogenetic analyses were carried out on the PCR-positive samples that were sequenced using Sanger protocols. Understanding the epidemiology of these viruses is essential for evaluating the implementation of effective prevention strategies.

## 1. Background

Over the past few decades, equestrian sports, both for competitive and leisure purposes, have met impressive growth worldwide [1]: it is estimated that, only in Europe, the equine industry accounts for around 7 million horses [2]. Physical injuries are not the only pathologies that hamper the sporting career of a horse and the economic interests of owners: the sport–horse industry requires their frequent transportation, environmental changes and social group disruption, which are renowned stress-inducing immunosuppression factors for these animals [3,4,5,6]. This is together with potentially promiscuous environments, where the risk of exposure to animals with an unknown health status a situation that can favor the onset of new infections [7,8]. Therefore, considering frequent and even international movements for events such as auctions, sports competitions and breeding purposes, the prevention of infectious disease outbreaks in these animals has become of paramount importance [9,10,11]. In addition, pathogens might be relatively new and not even part of a routine screening program, making it difficult to estimate their effective prevalence and morbidity levels, especially when their outcome is mainly asymptomatic, thus allowing them to go unobserved and, therefore, assisting in their diffusion. In fact, in recent years, a variety of equine viruses have gained greater importance as emergent and re-emergent viruses, such as: the *Equine coronavirus* [12,13], *Equine hepacivirus* [14], two Equine Pegiviruses (*caballi* and *equi*) [15,16,17], *Equine parvovirus-hepatitis* (EqPV-H) [18], *Equid hepatitis B virus* [19], and Equine Rotavirus B (ERVB) [20], underlining how vast and still unknown the range of infectious pathogens is in equids.

Considering the limited information regarding their presence in Italy, the biomolecular prevalence of two Pegiviruses affecting equines (*caballi* and *equi*) and of the *Equine coronavirus* was verified in two distinct Italian horse populations. Positive PCR amplicons were then sequenced using the Sanger protocol for the phylogenetic study of the detected isolates.

## 2. Introduction

Pegiviruses are small (8.9–11.3 kb), positive-sense, single-stranded RNA viruses and their genetic structure is similar to that of other Flaviviridae members [21,22]. Currently, the genus consists of 11 species [17] that infect a wide range of hosts such as: humans (a comprehensive review by [23]), non-human primates [24,25,26], bats [27,28], rodents [28,29,30,31], pigs [32], felines [33] and horses [15,16,34,35,36,37,38,39,40]; recently, new species were reported, such as a novel bottlenose dolphin *Pegivirus* [41], the new non-mammalian Pegiviruses infecting geese [42] and other birds [31], thus extending the spectrum of susceptible hosts.

Horses can be affected by *Pegivirus caballi* and *equi*. *Pegivirus caballi* was described for the first time in 2013 [15]. Although mainly asymptomatic, there is evidence of a slight increase in the liver enzymatic activity related to this infection [15,34,40] that deserves more in-depth analysis.

*Pegivirus equi* was previously known as Theiler’s Disease-Associated Virus (TDAV). It is a highly divergent virus, a member of the Flaviviridae family and was formally discovered in 2013 [16] while performing massive parallel sequencing to search specifically for the potential viral cause of TDAV. In fact, TDAV is one of the most common causes associated with acute serum hepatitis in horses and can lead to severe hepatitis with fulminant hepatic necrosis. It is usually associated with the administration of equine blood products, but its etiologic agent remained unknown for years [43]. The association of this *Pegivirus* with TDAV, however, was recently ruled out [22,44,45] and this disease is more likely related to the newly discovered EqPV-H [18]. Therefore, even if both Pegiviruses were initially associated with liver disease, recent data support the possibility that they are bone-marrow-tropic and non-pathogenic or, at least, their pathogenicity is not yet completely understood [22,43]. Overall, given the often subclinical development of the infection and the absence of a clear disease association, more studies are required to understand the pathogenicity and the implications of a long-term outcome of the infection [22].

The biomolecular prevalence range of *Pegivirus caballi* oscillates worldwide between 1 and 32% [15,16,34,35,36,38,39,40]; while after its official discovery, *Pegivirus equi* has instead been limitedly reported [16,40], being a contaminant of equine-derived commercial serum, along with *Pegivirus caballi*, [37,46,47,48] making these biological products the most feasible transmission specimen [16]. Other studies are required to verify other possible natural transmission routes [49].

Regarding the *Equine coronavirus* (family: Coronaviridae, genus: *Betacoronavirus*) (hereon *ECoV*), this shares the same characteristics of other Coronaviridae viruses, being a large, non-segmented, enveloped and single positive-stranded RNA virus of approximately 30,500 nucleotides in length [50,51,52]. Betacoronaviruses also infect bovines [53,54], pigs [55,56], rodents [57,58], bats [59] and humans (a comprehensive review by [60]).

The *ECoV* was first described in 1999 in foals with enterocolitis and diarrheic episodes [12,13], but it was genetically characterized only recently [61,62]. In recent years, numerous cases were reported worldwide: *ECoV* was detected both in healthy and in symptomatic horses [50,51,63,64,65,66,67,68,69,70,71,72,73] and, on the whole, the role of subclinically infected animals as virus spreaders seemed to be highly relevant [71]. In addition, seasonal trends were also highlighted that were not attributed to a specific cause [70,74,75,76]. *ECoV* infections were reported both in foals and in adult horses and predominantly affect the gastrointestinal apparatus, causing clinical signs, such as lethargy, weight loss due to diarrhea and colic, apathy and fever as the disease progresses (a comprehensive description in [77]), while respiratory symptoms [64] and neurological disorders [63,78,79] are rare. However, the infection has generally a benign course with low fatality rate cases [63,73,75,78]. Experimental infections were successfully performed, both leading to symptomatic and asymptomatic outcomes [80,81,82] and highlighting the oral–fecal transmission as the main natural route of the virus [37,81], possibly due to poor hygiene or a lack of individual space in shared environments. Nonetheless, *ECoV* RNA was detected also in respiratory secretions, making nasal secretions another potential source of virus transmission [68,82,83].

Overall, *ECoV’s* worldwide biomolecular prevalence ranges from <1 to 28.5% [50,64,65,67,68,69,83], with an even higher prevalence when stable outbreaks are reported [63,71,84]. However, these data are not available for Italy and for this, a study was undertaken in this context in two populations of national importance, represented by the equid population of the Italian Armed Forces (IAF) which add up to a number where a reliable prevalence can be estimated for the viruses under study.

## 3. Materials and Methods

### 3.1. Study Population

**Figure 1:**
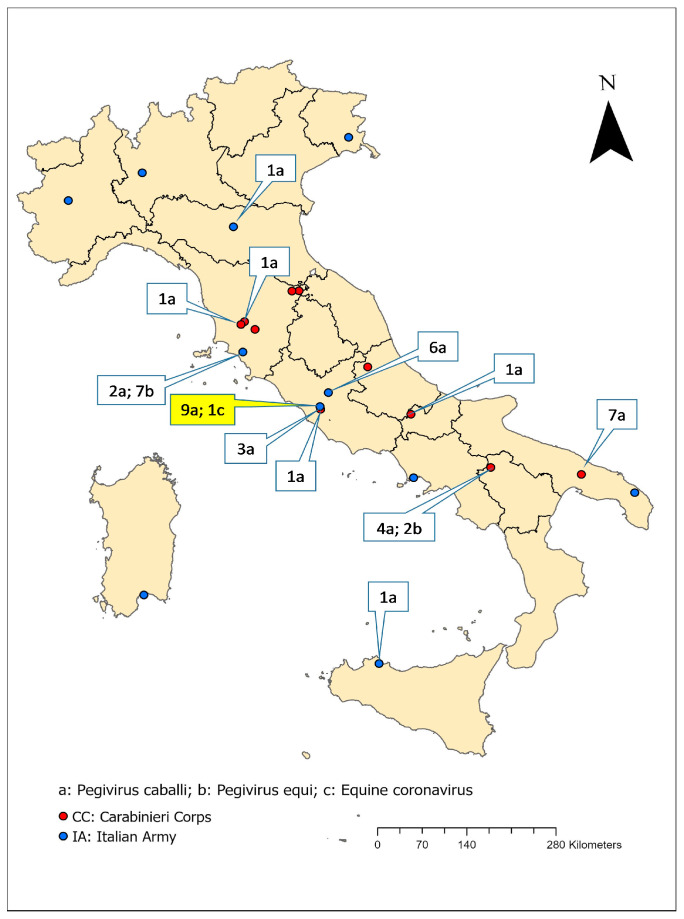
Geographical distribution of the stables considered for this study: red dots represent CC stables, while blue dots, IA stables. Region borders are reported. Tags represent type of positive samples and relative number: a: *Pegivirus caballi*; b: *Pegivirus equi*; c: *Equine coronavirus*. The yellow tag represents the stable also positive for *Equine coronavirus*.

The sampling focused on the equine population belonging to the IAF consisting of 1070 animals, with 401 horses belonging to the Italian Army (IA), mostly the Sella Italiano breed (94%), and 669 horses of the Carabinieri Corps (CC) where the Maremmano and the Murgese breeds were the most frequent (41% and 31%, respectively). At the time of the sampling though, the sampling population was represented by 372 and 671 individuals, respectively, for the IA and for the CC. A simple random sampling method was defined, considering an unknown prevalence (50%), a desired precision of 5% and a confidence level of 95%. It was used to select the number of individuals to be analyzed for each infection (Pegiviruses and Equine coronavirus) and biological sample (serum and rectal swab): the final number of samples required to estimate the biomolecular prevalence of the studied viruses was, respectively, 198 for the IA and 238 for the CC. A blood serum sample and a rectal swab was collected between 2021 and 2022 for each horse enrolled in this study. The samples were originally collected, respectively, within the equine infectious anemia national surveillance plan and parasitological monitoring activities conducted by the IAF. The individuals enrolled in this study were those with both target samples. The leftover biological material (both sera and rectal swabs) was aliquoted and stored at −80 °C for further investigating the presence of viruses within the aim of this study. The national geographical distribution of the stables is described in Figure 1.

As the distinct sample sizes were calculated for each biological specimen and for each of the IA and CC horse populations to be, respectively, screened for the equine Pegiviruses and *ECoV*, individuals enrolled were not necessarily overlapping for the analysis of the different viruses.

### 3.2. Extraction and Molecular Detection of Viral RNA

RNA extraction was performed using the QIAsymphony DSP Virus/Pathogen Kits by the automated extractor (QIAsymphony SP, QIAGEN, Valencia, CA, USA) following the manufacturer’s indications.

#### 3.2.1. Molecular Detection of *Pegivirus caballi* and *equi*

The 5′UTR fragment was chosen as the target for the *Pegivirus caballi* and *equi* PCR protocol [37], with primers amplifying a conserved region, common to both viruses and present in both reference sequences KC410872 [15] and KC145265 [16], as well as including two probes capable of distinctly detecting the two viruses within the portion amplified by the same primer pairs. The sequences of the primers and probes used are described in Table 1.

A duplex Real-time RT-PCR was set up, modifying the triplex Real-time RT-PCR designed by [37] using the QuantStudio™ 7 Flex Real-Time PCR System (Applied Biosystem, Waltham, MA, USA) and the Luna Kit Mastermix (Luna^®^ Universal Probe One-Step RT-qPCR Kit) with final primer and probe concentrations, respectively, of 0.4 µM and 0.2 µM. The Real-Time RT-PCR conditions were incubation at 55 °C for 10 min, followed by 95 °C for 1 min and 45 cycles at 95 °C for 10 s and 60 °C for 1 min. The synthetic reaction positive controls were designed for both viruses using the target amplified by the primers discriminating the two Pegiviruses.

#### 3.2.2. Molecular Detection of *ECoV*

For the *ECoV* detection, a Real-Time RT-PCR was set up (QuantStudio™ 7 Flex Real-Time PCR System (Applied Biosystem, Waltham, MA, USA)) using the Luna Kit Mastermix (Luna^®^ Universal Probe One-Step RT-qPCR Kit, New England Biolabs Inc., Ipswich, MA, USA), as well as primers and probes targeting the N gene of the virus, as already reported in [63]. A synthetic reaction positive control was designed specifically. The Real-Time RT-PCR conditions were incubation at 55 °C for 10 min, then 95 °C for 1 min, followed by 45 cycles at 95 °C for 10 s and 60 °C for 1 min. Details of the sequences of the primers and probes used are available in Table 1 below.

### 3.3. Sequencing

Two conventional RT-PCR assays, as described below, were set up for the genetic analysis of the Real-Time RT-PCR-positive samples through Sanger sequencing.

#### 3.3.1. Sequencing of Pegivirus-Positive Samples

Primers from [37] were chosen to perform a conventional PCR to detect a conserved part in the NS3 coding region common to both Pegiviruses and to confirm the duplex Real-Time RT-PCR positive samples. The expected size of the amplicon was 355 base pairs (bp). The primer sequences of the chosen primers are described in Table 1.

#### 3.3.2. Sequencing of *ECoV*-Positive Samples

Previously reported primers [85] were selected to set up a nested RT-PCR protocol to amplify the *ECoV* Real-Time PCR-positive samples. The target was the RNA-dependent RNA polymerase (RdRp), a highly conserved region of the ORF1ab polyprotein gene, and the expected size of the amplicon was 440 bp. The primer sequences are in Table 1.

#### 3.3.3. Sequence Analysis

The correct amplification of the PCR products was verified by capillary electrophoresis (QIAxcel Advanced, QIAGEN, Hilden, Germany). The amplification products of these PCRs were purified following manufacturer’s instructions (ExoSap, Applied Biosystems by Thermo Fisher Scientific, Santa Clara, CA, USA) and purification was checked by another capillary electrophoresis (QIAxcel Advanced, QIAGEN, Hilden, Germany). Samples which come out as a clean band were considered eligible for sequencing. The sequencing procedure was performed on Sanger Sequencing 3500 Series Genetic Analyzers (Applied Biosystems, Waltham, MA, USA) using a BigDye sequencing Kit V3.1. Sequences were assembled and analyzed using a Tracy pipeline (v. 0.7.3).

**Table 1:** The sequences of the primers, probes, PCR targets and bibliographical reference of the biomolecular investigation methods applied in the present study are reported in the table below.

For	Reagent	ID	Sequence 5′–3′	Target	Purpose	Ref.
Equine Pegiviruses	Primers fw	EVT-146	AGGGTTCTTCGGGTAAATCC	5′UTR	Duplex real-time RT-PCR	[16,37]
	Primers rv	EPgV-314	TCGKCGAGCYACAGACCGT	5′UTR	Duplex real-time RT-PCR	[37]
	Probes	EPgV1WST-189	6FAM-TGTTGTGATTGTGTTAGGGCAGGTGGCA-BHQ-1	Pegivirus caballi 5′UTR	Duplex real-time RT-PCR	[37]
	Probes	TDAV-199	TEX-TGTTTTGGGTTCAGGGCAGTAG-BHQ-2	Pegivirus equi 5′UTR	Duplex real-time RT-PCR	[37]
Equine Pegiviruses	Primers	TDAV-3744 Fw	GGAGCCCGGAGGGCATGGGTA	NS3	Conventional PCR	[37]
	Primers	TDAV-4098 Rv	TGGCAGGGACAAGGGTGGACT	NS3	Conventional PCR	[37]
ECoV	Primers	ECoV-380f	TGGGAACAGGCCCGC	Gene N	Real-Time PCR	[63]
	Primers	ECoV-522r	CCTAGTCGGAATAGCCTCATCAC	Gene N	Real-Time PCR	[63]
	Probes	ECoV-436p	6FAM-TGGGTCGCTAACAAG-TAMRA	Gene N	Real-Time PCR	[63]
ECoV	Primers Ext-Fw	Primers Ext-Fw	AAATTTTATGGCGGCTGG	ORF1AB	Nested-PCR	[85]
	Primers Ext-Rv	Primers Ext-Rv	GGACCTCATGAATTCTGTTC	ORF1AB	Nested-PCR	[85]
	Primers Int-Fw	Primers Int-Fw	GGTTGGGATTACCCTAAGTGTGA	ORF1AB	Nested-PCR	[85]
	Primers Int-Rv	Primers Int-Rv	ATGATGATTTTGAGTGATGATGG	ORF1AB	Nested-PCR	[85]

### 3.4. Phylogenetic Tree Construction

The partial sequences of the NS3 gene of *Pegivirus caballi* and *equi* were used to infer phylogenetic relationships with other most similar sequences, downloaded from the Genbank database [86]. Online webtool BLAST (https://blast.ncbi.nlm.nih.gov/Blast.cgi, accessed on 10 February 2025) was used for the comparison of all the sequences obtained.

Multiple alignments were performed using the Muscle web tool (https://www.ebi.ac.uk/jdispatcher/msa/muscle?stype=protein, accessed on 20 February 2025) and the corresponding output was used for building the Maximum Likelihood nucleotide phylogenetic tree.

IQ-TREE software (v. 2.1.4-beta) was used for finding the best substitution model and the phylogenetic tree inference was carried out by using the reference sequence of bat *Pegivirus* G (KC796076), representing the outgroup for the Pegiviruses tree. The phylogenetic tree was generated with a bootstrap value of 1000 and the figure was created using functions of the ggtree R library [87].

## 4. Results

### 4.1. The Sampling Population

The number of samples analyzed, both sera and rectal swabs to estimate the biomolecular prevalence of the studied viruses, was respectively 198 for the IA and 238 for the CC. At the end, 308 different individuals for IA and 381 for CC were analyzed, as 88 of IA and 95 for CC horses were randomly selected for both specimens. All the horses were reported as healthy during the entire duration of the sampling.

### 4.2. Extraction and Molecular Detection of Viral RNA

RNA extraction was carried as described above and the RNA detecting thresholds were set as follows: samples were ultimately considered PCR-positive if scoring a cycle threshold (Ct) lower, or equal, to 38 in the final run and that were, therefore, confirmed as PCR-positive at least twice. The used positive controls were dsDNA synthetic standards for the PCR assays (210 base pairs, 300 ng). A LOD curve (limit of detection) was performed for each standard and the target copies range covered from 1.303 × 10^12^ (the starting dilution) to 1.303 × 10^2^ (Ct = 40). Therefore, samples were considered PCR-positive if scoring a Ct ≤ 38, which accounted for around 1.303 × 10^3^ target copies per reaction, to guarantee the efficiency of the assay.

Each analyzed sample that tested PCR-positive, doubtful (Ct = 39–40) or close to the positive detection limit in the first PCR run (screening) was run a second time (confirmatory run) to evaluate the possibility of cross contamination or false-positive results. Samples that scored Ct > 38 in the first run were not confirmed in the second run and were registered as negative. All samples with Ct ≤ 38 were confirmed instead and registered as PCR-positive. Concerning the clear negative samples, instead, horse sera that tested PCR-negative did not give any PCR signal, which allowed a simple and neat discrimination between positive and negative samples in any sample that did give a PCR signal instead.

#### 4.2.1. Molecular Detection of *Pegivirus caballi* and *equi*

PCR-positive samples were detected for both the IA and the CC horses: for the IA, 13 samples tested positive for *Pegivirus caballi* and seven for *Pegivirus equi* while for the CC, 24 samples tested positive for *Pegivirus caballi* and two for *Pegivirus equi.*

Intrapopulation PCR-positive percentages were for the IA: 6.6% (13/198-95% CI: 3.5–11.0) for *Pegivirus caballi* and 3.5% (7/198-95% CI: 1.4–7.1) for *Pegivirus equi*.

For the CC: 10.1% (24/238-95% CI: 6.6–14.7) for *Pegivirus caballi* and 0.8% (2/238-95% CI: 0.1–3.0) for *Pegivirus equi*.

#### 4.2.2. Molecular Detection of *ECoV*

Only one sample belonging to the CC horses was *ECoV* PCR-positive (0.4%, 0.0–2.3%; 1/238).

### 4.3. Phylogenetic Analysis

All Real-Time RT-PCR-positive samples were analyzed using conventional PCR to confirm the positivity for each virus and to obtain a fragment suitable for sequencing.

Of the overall 45 positive amplicons (44 for Pegiviruses and 1 for *ECoV*), eleven were successfully sequenced for equine Pegiviruses, together with the only one positive amplicon for the *ECoV*: the obtained fragments were uploaded on GenBank (OR166386; OR772901-910) (see Table 2 below for details).

### 4.4. Phylogenetic Tree Construction

**Figure 2:**
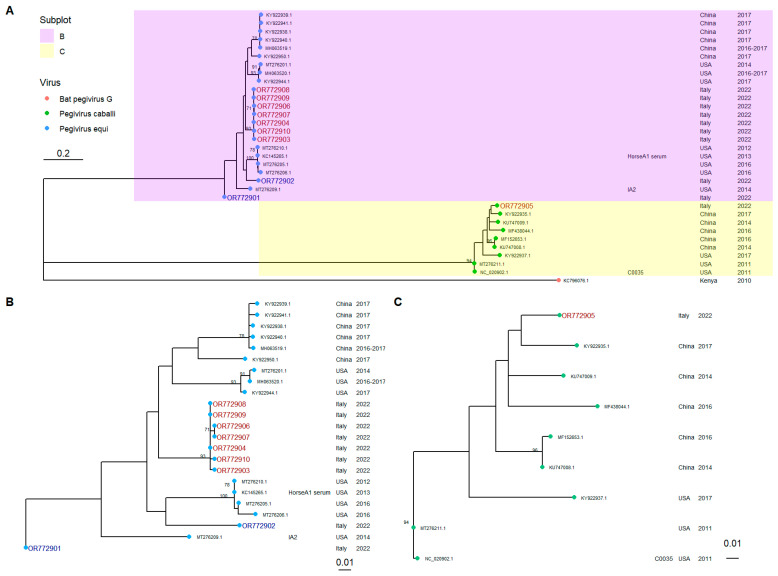
Phylogenetic tree of partial sequences of NS3 of *Pegivirus caballi* and *equi*. (**A**) depicts the phylogenetic tree including both viruses and nodes corresponding to *Pegivirus caballi* and *equi*, which are highlighted in yellow and purple. (**B**,**C**) show more detailed subtrees of *Pegivirus caballi* and *equi*, respectively. Colors of tips show a simplified viral taxonomic classification instead of that reported in NCBI. Each round tip is described by information about the reference isolate, country and date of collection. Blank space equal to unavailable information. The accession numbers of Italian samples belonging to CC are painted blue, whereas those of IA are red. Only clades with a supporting bootstrap value greater than 70 are shown. The outgroup is the reference sequence of bat *Pegivirus G* (KC796076).

The sequences of the Pegiviruses detected were used to build a phylogenetic tree (see Figure 2).

The phylogenetic analysis of equine Pegiviruses, carried out with the Maximum Likelihood method log likelihood (−2380.4574), giving the best substitution model that is GTR+F+G4 (BIC score 5185.3118), was built from a 363 bp long multiple alignment involving nucleotide sequences of 33 isolates. Sequences belonging to *Pegivirus caballi* are distant from those of *Pegivirus equi*, as confirmed by the high bootstrap value of its external node (94%). Figure 2B depicts the group of *Pegivirus equi* more specifically and among those detected during this study, the ones belonging to the IA stand together within the same group, as confirmed by the high bootstrap value of the node (93%), whereas positive samples of the equids owned by the CC did not gather in the same group of the sequences of the ones of the IA, even if these nodes were not well supported by bootstrap values. Nucleotide alignment locates the sample OR772901 at a more external node of the *Pegivirus equi* whole group, despite the fact that, for the tool ‘Identical Protein Group’ of NCBI, its protein sequence (WPE03500.1) is identical to the sequences of the IA (WPE03503.1, WPE03505.1, WPE03506.1 and WPE03508.1). In addition, by aligning its amino acid sequence with the others of the IA (WPE03501.1, WPE03502.1, WPE03507.1 and WPE03509.1), we obtain a 100% identity, as shown in Table 3. Furthermore, Table 3 depicts the result of the protein alignment against the reference *Pegivirus equi* sequence (Horse A1 serum, AGH70217.1), where the only difference is represented by a substitution of an S with R (S1046R considering the position of reference sequence AGH70217.1). This kind of substitution is shared with all the CC and IA sequences; however, the corresponding position in WPE3502.1 (OR772903.1) has not been obtained.

On the other hand, Figure 2C’s depiction is more in-depth, with the resulting group represented by *Pegivirus caballi* sequences, even if the topology of this branch is not well supported by the high bootstrap values. In this case, the protein alignment between the IA sequence (WPE03504.1, corresponding to OR772905.1) and the protein sequence of the reference isolate C0035 (YP_007697649.1) exhibits 98% identity with a query coverage of 100% with two substitutions occurring, T1115A and S1117R, considering the reference position as that of the C0035 isolate.

For *ECoV*, the result of the alignment of OR166386 against several known *ECoV* isolates is shown in Table 4, revealing that the difference among the isolates for that region of Rdbp is less than 1%.

## 5. Discussion and Conclusions

Both new and re-emerging diseases can represent a threat to equestrian sports and selective breeding in horses, activities that represent a solid economic business worldwide, especially when transmission routes are still elusive, without the possibility of adopting specific preventive measures. Among the newly identified Italian *Pegivirus equi*-submitted sequences, those belonging to the CC do not cluster together with those of the IA, and the sequences of the latter group up all together. In addition, one sample of the CC group stands at the more external node of *Pegivirus equi*, even if the protein sequence of this isolate is identical to those of some Italian IA sequences, indicating that probably there had been synonymous mutations. For the protein sequences of the *Pegivirus caballi* and *Pegivirus equi* isolates detected in this study, there are, respectively, two and one amino acid substitutions when compared to their respective reference isolates (C0035 and Horse A1 serum). These substitutions do not seem to be involved in any active sites, when considering the Hepacivirus NS3 protein (PROSITE accession PS51822 and ProRule PRU01166). In fact, little is known about the active sites of the NS3 protein in *Pegivirus caballi* and *equi*; however, considering also another study on human *Pegivirus* (GB virus C) [88], Ser1062, which is an active site of the NS3 protein, is conserved among all our samples.

For *ECoV*, as shown in Table 4, the in silico analysis suggests that there is only a slight difference among the majority of *ECoV* samples, including NC99, CH21, Tokachi09, Obihiro12-1 and Obihiro12-2. Moreover, [71] indicates that the structural proteins are highly conserved and in Figure 1 of [89], it can be seen that the least similar region might be that of the NS2 coding gene.

Although further studies are essential to obtain more information about these viruses and their clinical role, the data presented here describe the first study on the biomolecular prevalence of *Pegivirus caballi* and *equi* and *ECoV* in specific horse populations present in Italy. In fact, the only information available confirming the presence of these viruses in this territory is from [37] in the form of pooled biological products, originating from Italian donor horses testing *Pegivirus caballi*- and *equi*-positive. Therefore, in view of the limited knowledge available on these Pegiviruses, as well as for ECoV, our data are especially relevant and useful to improve and update the circulation of equine emerging viruses.

In fact, the observed presence for these viruses on a biomolecular level confirms their recent or active circulation within horse populations. The biomolecular prevalence obtained for *Pegivirus caballi* of 6.6% and 10.1%, respectively, for the IA and CC highlights how these horse populations have come into contact with both viruses, remaining within the range already observed and reported worldwide which fluctuates between 1 and 32% [15,34,35,36,37,38,39,40,46]. For *Pegivirus equi*, instead, the prevalence obtained are lower: respectively, 3.5% and 0.8% for AI and CC. These data are, however, in line with a previous paper [40] where the described prevalence for this virus was 1.6% (Brazil).

Although the clinical relevance of both viruses is still being debated, and up to now, it seems to be minimal, the present data confirm their active circulation in Italian horses between 2021 and 2022, and referring to the horses detected as positive in this study, for all, a clinically healthy status was referred.

In fact, even though the pathogenic outcome of infection due both Pegiviruses (*caballi* and *equi*) is still to be evaluated, these viruses may still play a role in compromising the immune system of the infected individual, in consideration of their ability to cause persistent infection that cannot be easily cleared by the immune system [22]. Therefore, it cannot be completely ruled out that this would make the infected animals more susceptible to other infectious diseases, especially in immunocompromised subjects.

In addition, as currently only the iatrogenic transmission route has been demonstrated to be successful in spreading the virus [16,90], the frequent detection of these Pegiviruses in commercial biological products and serum pools [37,46,48] suggests that hemoderivates may play, or may have played, a major role in spreading the infections worldwide. In fact, the efficient development of infection and post-inoculation with infected biological products was reported for both *Pegivirus caballi* [90] and *equi* [16], as well as experimentally using full-length molecular clones [22].

In view of this, for biosecurity reasons, guidelines of diagnostic methods aimed at the detection of newly emerging and re-emerging infectious agents, representing threats in blood-derived biological products, as well as methods for their inactivation, should be constantly updated to guarantee the innocuity and biological safety of these products. As the main clinical outcome of both Pegiviruses is usually subclinical, the carrier status could represent a risk for other horses. The fact that the PCR-positive animals were apparently healthy confirms the need to monitor for the presence of these viruses in consideration of the fact that natural transmission routes are essentially unknown and can be spread using different routes, as already confirmed, which is through the administration of blood products.

For *ECoV* infection, once suspected and due to its highly contagious nature, any horse that develops or shows significant fever, anorexia and depression, with or without enteric signs (such as colic or diarrhea), should be isolated until a diagnosis is made or the clinical signs disappear. In support of this, the prevention of this infection should focus on the implementation of routine management practices aimed at reducing the likelihood of the introduction and transmission of this pathogen in an equine facility [51].

All considered, for both *Pegivirus caballi* and *equi*, it would be advisable to include them in the panel of viruses to be tested in hemoderivates used in veterinary treatments and also consider these viruses together with *ECoV* in screening programs for horses subject to frequent movement, especially those of a high genetic value.

## Figures and Tables

**Table 2:** ID, property, sequencing result and accession number of sequenced positive samples are reported in the table. IA: Italian Army, CC: Carabinieri Corps.

	Sample ID	Horse Population	Virus Identified	Accession Number
1	50638/48	CC	*Equine coronavirus*	OR166386
2	8844/46	CC	*Pegivirus equi*	OR772901
3	8844/48	CC	*Pegivirus equi*	OR772902
4	75424/6	IA	*Pegivirus equi*	OR772903
5	75424/5	IA	*Pegivirus equi*	OR772904
6	84400/11	IA	*Pegivirus caballi*	OR772905
7	77362/13	IA	*Pegivirus equi*	OR772906
8	76019/16	IA	*Pegivirus equi*	OR772907
9	75424/11	IA	*Pegivirus equi*	OR772908
10	76019/22	IA	*Pegivirus equi*	OR772909
11	76019/23	IA	*Pegivirus equi*	OR772910

**Table 3:** Results of the alignment of WPE03500.1 against the non-identical Italian sequences and that of horse serum A1 (AGH70217). A.N. stands for Accession Number.

Protein A.N.	Query Cover	E-Value	Ident%	Nucleotide A.N.
WPE03507.1	100%	2 × 10^−87^	100.00	OR772908.1
WPE03501.1	99%	1 × 10^−86^	100.00	OR772902.1
WPE03509.1	97%	3 × 10^−85^	100.00	OR772910.1
WPE03502.1	97%	3 × 10^−85^	100.00	OR772903.1
AGH70217.1	100%	2 × 10^−77^	99.15	KC145265.1

**Table 4:** Results of the alignment of OR166386 against other known sequences of *ECoV*. A.N. stands for Accession Number.

Nucleotide A.N.	Query Cover	E-Value	Ident%	Isolate
LC061273.1	100%	0.0	99.49	Obihiro12-1
LC061274.1	100%	0.0	99.49	Obihiro12-2
MZ562881.1	100%	0.0	99.24	CH21 isolate Haribo
EF446615.1	100%	0.0	99.24	NC99
LC061272.1	100%	0.0	98.99	Tokachi09
KY458232.1	100%	0.0	98.48	
OL770366.1	100%	0.0	97.47	

## Data Availability

The datasets used and/or analyzed during the current study are available from the corresponding author on reasonable request.
